# Medicine And Money: Friends Or Foe?

**DOI:** 10.4103/0973-1229.27607

**Published:** 2006

**Authors:** Adamson S. Muula

**Affiliations:** * *Department of Community Health, University of Malawi, College of Medicine, Malawi and Department of Epidemiology, University of North Carolina at Chapel Hill, United States.*

**Keywords:** *Brain Drain*, *Physician Training*, *Medical Specialisation*, *Informal Payments*, *Conflict of Interest*, *Relation with Pharma.*

## Abstract

The relationship between medicine and money is a delicate one that all people involved need to handle responsibly. If one becomes a physician for the mere fact of pursuing money, s/he may soon find that another profession or activity may have fulfilled such a need in a better way. While in the practice of medicine the interest of the patient is paramount, this does not suggest that the welfare of the physician should be neglected at all. It is much about a balance of priorities between the legal and ethical pursuit of money and the promotion of the health of society. Physicians are not called to a life of self-denial, poverty and destitution, as others may be tempted to suggest. The service of patients and the honest reward from one's labour are not incompatible. Where conflict of interest arises, it is prudent for a physician to always remember never to harm oneself, the profession and the patient more than what can be gained.

## Introduction

It is generally accepted that the relationship between the physician and the patient is a fiduciary one where the latter's interests should supersede those of the physician (Jones *et al*, 2005). Probably we should be saying that such was the ‘traditional’ perception of both physicians and the general society. There are certainly physicians who, in altruistic ways, consider the interest of society as the primary objective of their professional practice. Moreover, some think that the primacy of the interests of patients is not in line with a life of living in obvious wealth i.e. medical practice is thought of on the lines of sacrifice, not as means to earn a living. It is argued that the practice of medicine is a vocation, a calling, or some sort of priesthood.

There is also a perception that medicine is just like any other profession. If lawyers and accountants are making money from their sweat, what is wrong with physicians earning an enviable life? Why are people not worried about how affluent lawyers become, but do quickly criticize if physicians seem to be getting a fair wage?

This is a health debate that should be encouraged and nurtured. There may not be clear-cut solutions to such complex issues. But certainly there will be enlightenment for all of us, and we are likely to be able to appreciate our own, and other people's points of view, from such a discourse.

## Complexity of Training

There is probably no other profession as yet whose training takes as long as that of becoming a physician. Being a specialist physician is even worse (Ramesh, 2005). It can take up to about eight years to a decade while one is still in training. In fact, the training never ends. Although many physicians do love the continued medical education that is associated with the profession, it can also be safely said those who dislike lifelong learning but are physicians certainly have found themselves in a profession they should get out from. Continued professional development is not so much a matter of choice, as a matter of duty. Or else mediocrity creeps in and such a physician's practice becomes a danger to society. Continued education is a requirement, even when it is ‘self-imposed’, that essentially every doctor must have in order to be relevant in an ever-changing world of clinical practice.

The cost of medical training is comparably higher than of any other professional training. In many universities, liberal arts and social sciences courses normally have the lowest tuition fees while science courses (physics, biology, biochemistry) require much higher tuition fees. Medical students, who are basically receiving a mixture of science and arts, have to pay higher tuition fees than any other students. In situations where students and their families are expected to pay the fees at market rates, there are expectations, and justifiably so, that once the student graduates as a doctor, s/he should be able to recoup the investment in training. Low remuneration associated with the practice of medicine in many of the least developed nations of the world is therefore a disincentive for the physician to continue working in their home countries (Garg, 2005).

## Corruption In Medicine

Although corrupt tendencies have been reported frequently among politicians, the medical field has not been spared from this vice (Ausman, 2005). There are several motivations for the practice. Not attempting to justify any of these motivations, I will just highlight some of the reasons provided as to why corruption does occur in medicine. These include poor remuneration, greed and ignorance.

### Poor Remuneration

The importance of remuneration in keeping a functioning health system has attained particular importance mostly in sub-Saharan Africa with regard to what has been described as the ‘brain drain’ of health professionals (Chandra, 2006; Dovlo, 2005). Among the many shortcomings of the health systems that are ‘losing’ physicians and other health professionals to developed nations, lack of adequate remuneration is one of the important reasons. Lack of money is therefore a significant push factor from the developing nation while presence and accessibility of money, in the recipient nations, is an attractive pull factor.

As stated above, the reasons for the shortage of health professionals in developing nations are many. There are problems with the actual and, in many instances, unrealistic numbers of health professionals being trained, who are far below the required numbers. The problem stems from poor investments in the training of health professionals across the globe. The push, pull and grab factors for the movement of health professionals have been described elsewhere (Muula, 2005, Paradath *et al*, 2003). The financial differentials among countries and regions of the world have facilitated the movement, as health professionals gravitate from resource-poor nations towards the developed ones. Several developed nations, especially Australia, United States, and the UK are unable to deliver adequate health services without international medical graduates. This situation is likely to get worse as the population of the developed nations continues to age, and need for health services escalate, while investment in the training of physicians does not match up to the growing need.

Responses to the global “brain drain” can be varied. ‘Let it happen’. ‘Let us regulate it’. ‘Let us prevent it’. ‘Let us promote it: after all it is just brain circulation and it is a free world’. Each one of these possible approaches has strengths and weaknesses.

### Let It Happen

Deciding just to let the brain drain or the movement of health professionals mainly from the developing nations to developed world continue, fails to appreciate the fact that many physicians from the developing nations are trained using tax payers′ money. These countries are training people with the hope that they will contribute to service provisions in their home countries. A loss of a physician to the developed nations is several thousand United States dollars and years invested by a poor country gone down the drain. In the meantime, the developing nation is convincing itself that physicians do not really matter to deliver health care, as other lower cadres health workers can deliver the same level of care that physicians can. This is happening when it has not been thought as to why developed nations do not invite the lower cadre professionals for health delivery in their own countries. There is this international mentality, which promotes the thinking that developing nations with inadequate supply of medicines, and crumbling health delivery systems, will benefit more from lower level health practitioners. The physician is too sophisticated for such an environment. Little thought is spared to consider whether the physician, who is better trained than the other lower cadre health professionals, would be the right person to contribute to solving the enormous health services problems being faced.

### Let Us Regulate The Flow

There have been attempts to regulate the flow of health professionals from the developing nations to the developed nations. Developed nations themselves ensure that immigrants have an acceptable level of knowledge, skills and competence. This however works for the benefit of the receiving country as this ensures that many of the physicians who leave southern countries may be among the cream of their countries. The recent UK policy where a doctor from outside the European Union seeking training post may not be admitted in the country unless it is shown that there was no other doctor within the Union to take up the post will probably have an impact on the migration of doctors from the developing world into the UK (UK Home Office, 2006). The policy has both its positives as well as negatives. On the positive side, the developing world may not lose as many of its physicians as has been the case in the past. However, the UK, although a major recipient of developing world physicians, is not the only destination. Other countries will continue to absorb them. On the negative side, it cannot be denied that developing world doctors had opportunities to obtain specialist qualifications in the UK before returning to their home countries. This opportunity has been threatened by the change of policy. Also developing countries physicians, who felt oppressed by poor work conditions in their home countries, have one option of a better life closed for them with this new policy.

There is a temptation to prevent the flow of health professionals. Donor (in this case, developing) countries would find this approach attractive. However, the flow of health professionals is happening not just because developing world health professionals see the grass greener in the West. There is a real need, and in fact growing appetite, for health workers in the West. Politicians in northern countries are being asked by the electorate as to what they are going to do with the increasing waiting times for elective procedures.

### Let Us Prevent It

The other option is: let us prevent it. Should doctors leave their posts in poorly remunerated jobs in search of higher paying situation elsewhere? Does it mean that physicians who are leaving developing nations to move to the developed world are less patriotic than those who remain in their countries of birth and training? These questions should also be complemented with somewhat different questions. Is it acceptable for a country to fail to pay its physicians remuneration commensurate with their input to the social sector? Are physicians′ families expected to suffer from economic neglect because their bread-winners cannot obtain the requisite finances for a comfortable life? Should physicians be prevented from leaving their countries of birth to practice in another land while other professions are not so prevented?

There is no doubt that unless the developing nations put their money where their mouth is, the brain drain situation, especially in sub-Saharan Africa, is not only going to occur, but likely to become worse. Of course money, of its own, may not solve all the problems. But without it, only little gains are likely to be achieved with non-monetary initiatives. And what are those initiatives that are not going to need money?

## Informal Payments

Informal payments, which can be legal, not so legal and clearly illegal, are an important source of income that has been documented in the literature on health policy and health human resources. Physicians across the world do receive informal payments. As a state tolerated practice, informal payment systems are common in many of the Eastern European transition countries of the former Soviet Union.

There are varying definitions of what informal payments are. In Hungary, it is defined as what patients pay directly to doctors for service they would normally receive for free in state health facilities (Gaal *et al*, 2005). These payments may be perceived as ‘tips,’ or paying to express gratitude to the physicians for their care. In Poland, for instance, in the late 1990s, up to 50% of physicians′ income was estimated to have been obtained from informal payments by patients to the doctors. (Chawla *et al*, 1998.) In some situations, informal payments are more like bribes than tips. They have nothing to do with a sense of gratitude or ‘thank you’. They are just outright bribery.

Medical research is a way of living for many medical doctors. Some researchers have been corrupted by greed and unethical pursuit of money. The desire for money, even more money, cannot be evil on its own. It is when people desire to acquire money through *questionable* means that the profession's reputation is put in jeopardy. And this reputation must be guarded zealously. However, as Ausman (2005) has suggested, corruption in medicine could be a symptom of what the wider society is going through. While this does not justify corruption in medicine, all it means is that reparative processes will have to be set up at another level.

## Medical Research

Different types of research, whether bench, clinical or operational, are important aspects of the delivery of health care services. Oftentimes, while the public is prepared to pay for actual costs of patient care, it becomes problematic when health centres plan to spend significant amounts of their income on research. Research may, unfortunately, be seen as not directly impacting on patient care. It is what academics do. In some settings, to be an academic simply means to be theoretical, not interested in real-life problems. In such situations, allocating money to research, which may have to compete with core clinical services, is considered a waste of resources. Not often do people realise that a health service without adequate attention to research is certainly on the path to poor quality services.

While many national research institutes may be funded from the tax-payers′ money, a significant contribution towards research is from the pharmaceutical industry. Creating and nurturing a healthy relationship between the practice of medicine and pharma can be a rewarding experience. Being unethically influenced by the profit interests of pharma is clearly a slippery slope we should all keep in mind and avoid as much as we can. However, realisation that a health system without constant inflow of knowledge from relevant research which affects that particular health system is suited for the morgue may tempt physicians, as others, to embrace pharma in questionable ways.

## Conflict Of Interest

Physicians are increasingly finding themselves in situations where potential and real conflicts of interest arise. Physicians have been shareholders in the pharmaceutical industry and hired to speak on behalf of pharma. Some physicians own various health care support services, such as medical laboratory services, radiology services etc. In physician-owned practices, it may be difficult for some to avoid over-treatment of patients. This can take various forms, but generally all involve providing additional services to patients not clinically indicated but with the potential to increase the health centre's income. This can involve over-prescription, prescription of non-generic medicines in order to generate more profit, performing procedures on patients that do not significantly change the clinical outlook of the patient but have high returns for the practice (Jones *et al*, 2005). Sometimes, physicians may fail to refer patients out for fear of losing income, although failing to refer jeopardizes the patients′ health, and often the long-term interests of the practitioner himself.

## Relationship With The Pharmaceutical Industry

The development of the art of medicine preceded the establishment of the corporate pharmaceutical industry. In the 21^st^ century, however, pharma is an integral part of modern medicine. However, the relationship between medicine and pharma has been that of ‘love and hate’.

The primary role of pharma is not to improve the health of anybody. It is to make profits for its shareholders. Contributing to a healthy society is the means by which such profits are generated. Period. With that in mind, the industry has various tools, sometimes described as ‘tactics’, aimed at befriending as many physicians as possible; and in so doing influence the prescribing habits of those physicians that it influences. Many scientific congresses and the consumables at congresses etc. are sponsored by pharma. However, it is prudent to note that there is just no free lunch.

It is of interest that the American Medical Student Association (AMSA) is running a “PharmaFree Campaign” aimed at refusing trips, meals and gifts from the pharmaceutical industry, as it is perceived that the industry cannot be trusted as a source of unbiased source of information (Moghimi, 2006). The AMSA PharmaFree Pledge is instructive:

I am committed to the practice of medicine in the best interest of patients and the pursuit of education that is based on the best available evidence, rather than on advertising or promotion. I, therefore, pledge to accept no money, gifts, or hospitality from the pharmaceutical industry; to seek unbiased sources of information and not rely on information disseminated by drug companies; and to avoid conflicts of interest in my medical education and practice (American Medical Students Association, 2006).

Finally, as has been suggested by Singh and Singh (2005-2006), it is time to do soul-searching and seriously consider if the practice of medicine should be:

i) Welfare-centred, ii) Corporate-oriented, or an iii) Patient welfare orientated enterprise. It is likely that a balance of all of these approaches may have to be embraced.

## Concluding Remarks

As early as the times of Hammurabi in Ancient Babylon, physicians had different ways of charging fees: ten shekels for a lifesaving procedure and only two if the patient was a slave (Gawande, 2005). Despite the central role that money has in the provision of health care services and medicine, the study of finances is an important omission in many medical schools. Either it is thought that issues with finances will take care of themselves, or that some other profession will take care of monetary issues while the physicians′ role is saving life. However, more lives could be saved if physicians maintain a healthy balance on how they view and deal with issues concerning money.

Medicine is at the crossroads. Much of what will be the nature of future practice depends on how the practitioners relate to money. This relationship has the potential to propel medicine to provide greater good to society, or be subsumed by personal interests, even greed, profiteering and a potentially suicidal self-interest.

## Questions That This Paper Raises

1. What will be the impact of the new UK Home Office's Immigration Requirements on the delivery of health care services in UK?
2. What will be the impact of the new UK Home Office's Immigration Requirements on developing world health systems?
3. Do you know of case studies of intervention aimed to retain health professionals in the developing world?
4. Are guidelines aimed at regulating physician-pharma interactions effective?
5. What are the other, as yet unrecognized, sources of financing in biomedicine, apart from pharma?
6. Does the stage of one's career and nature of employment (private practice, public hospital practice, university teaching centres, research institute, pharma), influence a physician's perception of pharma?

## About the Author


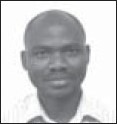


**Adamson Sinjani Muula** is a medical doctor and lecturer in community and Public Health at the Malawi College of Medicine. He graduated from the University of Malawi College of Medicine after studying at the Flinders University Medical School, Adelaide, Australia and Malawi College of Medicine. He is also a graduate in public health from the Loma Linda University and a Palliative Care graduate from Makerere University, Uganda. At the time of writing Adamson Muula was scholar at the University of North Carolina at Chapel sponsored by the AIDS International Training and Research Program (AITRP) at the Fogarty International Center, United States.

## References

[1] American Medical Students Association ((2006)). AMSA's PharmFree Medical Student Pledge.

[2] Ausman JI ((2005)). Corruption in medicine. Surgical Neurology.

[3] Chandra A ((2006)). The metrics of brain drain. New England Journal of Medicine.

[4] Chawla M, Berman P, Kawiorska D ((1998)). Financing health services in Poland: new evidence on private expenditures. Health Economics.

[5] Dovlo D ((2005)). Taking more than a fair share? The migration of health professionals from poor to rich countries. PLoS Med.

[6] Gaal P, Evetovits T, McKee M ((2006)). Informal payment for health care: Evidence from Hungary. Health Policy.

[7] Garg G ((2005)). Postgraduate lifestyle: stress and satisfaction. The Indian Journal of Pediatrics.

[8] Gawande A ((2005)). Medicine's Money Problem. The New Yorker Pact.

[9] Jones JW, McCullough LB, Richman BW ((2005)). Show me the money: The ethics of physicians’ income. Journal of Vascular Surgery.

[10] Moghimi Y ((2006)). The “PharmaFree” Campaign: Educating Medical Students about Industry Influence. PLoS Med.

[11] Muula AS ((2005)). Is there a solution to the “brain drain” of health professionals and knowledge from Africa?. Croatian Medical Journal.

[12] Paradath A, Chamberlain C, McCoy D, Ntuli A, Rowson M, Loewenson R ((2003)). Health personnel in Southern Africa: confronting maldistribution and brain drain. EQUINET Discussion Paper No. 3.

[13] Ramesh K ((2005)). Postgraduate entrance exams. The Indian Journal of Pediatrics.

[14] Singh AR, Singh SR, Singh SA ((2005-2006)). Where is Medicine Heading? Pointers and Directions from Recent Law Suits Against Industry: Medicine As A Corporate Enterprise, Patient Welfare Centered Profession, Or Patient Welfare Centered Professional Enterprise?. MSM.

[15] UK Home Office (2006) A Points-Based System: Making Migration Work For Britain.

